# Editor's choice: this December 2020

**DOI:** 10.4314/ahs.v20i4.2

**Published:** 2020-12

**Authors:** James K Tumwine

**Affiliations:** Editor in Chief, African Health Sciences

We have quite a number of papers in this issue and, honestly, I am lost for choice. None the less a few papers stand out for me.

In the infectious disease section, I was touched by Dr. Ombeva's sharing and reflection of his story: the way he caught Covid-19 from a child he was treating and the child's parents. 1 To me this is the story of the year and epitomizes what I have referred to as annus horribis. Then the Covid-19 vaccine story by Prof. Fikri and colleagues. 2

It is followed by a meta-analysis on seasonal flu that is highly relevant given that flu impacts the effects of Covid-19. 3

Then follows the attempt by the HIV community to understand the ramifications of the HIV pandemic in Africa despite having lived with it for almost 4 decades now! Hence we have papers on: outcomes on PLHIV in Kenya^4^ CD4 counts,^5^ VMMC and HIV risk in Uganda,^6^ HIV and fungal infection^7^, HIV in Wuhan China^8^ that city that gave Covid-19 to the world; and HIV and primary school teachers.^9^ Then comes dengue,^10^ hepatitis B and osteoporosis,^11^ TB,^12^ *H.pylori*,^13^ MDR Staph. aureus,^14^ antibiotics in Rwanda,^15^ adult pneumonia,^16^ *C.parvum*,^17^ antimalaria drugs^18^,^19^ and pulmonary micro-aspiration.^20^

## NCDS: our curse of the century

Even during this Covid-19 pandemic, NCDs are forever with thus: cardio-vascular disease nursing care,^21^ hypertention,^22^ HIV-exposed infants 23, ECG abnormalities in HIV,^24^ left ventricular hypertrophy,^25^ epicardial fat,^26^ aerobic exercise,^27^ insulin resistance,^28^ asthma,^29^ COPD and sleep,^30^ e cigarettes,^31^ non- alcoholic liver disease,^32^ home care,^33^ Bilateral paediatric surgery,^34^ self-harm,^35^ MRI,^36^ osteosarcoma risk,^37^ thyroid 38 and gastric esophageal cancer;^39^ fibromatosis,^40^ prostate cancer,^41^ neuro-endocrine tumour,^42^ polycystic ovary and vitamin D deficiency,^43^ micro RNAs and disease:^44^ all will hopefully make us appreciate the burden and effect of this scourge on our people.

## Reproductive and child health issues

In this section we have issues of pregnancy (ectopic,^45^ teenage, 46) labour,^47^ caesarean section,^48^ gestational age,^49^ home delivery,^50^ postpartum depression,^51^ female genital mutilation in the Horn of Africa^52^ and Nigeria,^53^ menopause and sexuality,^54^ infertility,^55^ hand washing,^56^ anaemia in children in Ethiopia,^57^ and breastfeeding efficacy.^58^

The rest of the papers are on self-medication,^59^ halitosis^60^ and trace elements toxicity. 61
